# Masked vitamin B12 deficiency in pernicious anaemia: A diagnostic trap due to assay interference—Case report

**DOI:** 10.1177/2050313X251377211

**Published:** 2025-09-15

**Authors:** Mohammed Abdulgayoom, Elabbass Abdelmahmuod, Abdulrahman F. Al-Mashdali, Yara R. Abuazab, Aliaa Amer, Ibrahim Ganwo, Shehab Mohamed

**Affiliations:** 1 Department of Haematology, National Center for Cancer Care and Research, Hamad Medical Corporation, Doha, Qatar; 2 Endocrine Section, Department of Medicine, Hamad Medical Corporation, Doha, Qatar; 3 Department of Family Medicine, Hamad Medical Corporation, Doha, Qatar; 4 Department of Laboratory Medicine and Pathology, Department of Hematopathology, Hamad Medical Corporation, Doha, Qatar

**Keywords:** megaloblastic anaemia, B12 deficiency, pernicious anaemia, anti-intrinsic factor antibody, assay interference

## Abstract

Megaloblastic anaemia is mainly caused by vitamin B12 or folate deficiency. However, rare cases present with discordant laboratory findings, particularly falsely elevated vitamin B12 levels due to assay interference from anti-intrinsic factor antibodies, complicating diagnosis and management. We describe a 64-year-old woman with macrocytic anaemia, pancytopaenia, and megaloblastic changes on the blood smear, yet with a markedly elevated serum B12 (>2000 pmol/L). A strongly positive anti-intrinsic factor antibody titre explained the paradoxical result. Empirical parenteral B12 therapy led to rapid haematological recovery, confirming masked B12 deficiency secondary to pernicious anaemia. This case highlights the critical pitfall of anti-intrinsic factor antibody-mediated assay interference in vitamin B12 testing, which can mask true deficiency. Clinicians must maintain a high index of suspicion for masked B12 deficiency in patients with megaloblastic anaemia and incongruent laboratory results, particularly in the presence of anti-intrinsic factor antibodies.

## Key clinical message

Patients with megaloblastic anaemia may show falsely elevated B12 levels due to assay interference by anti-intrinsic factor antibodies. Clinicians should not rely solely on serum B12 levels. A high index of suspicion, clinical judgement, and early treatment are crucial to avoid delays in diagnosing and managing true B12 deficiency.

## Introduction

Megaloblastic anaemia is a well-characterized haematological disorder resulting from impaired DNA synthesis, most commonly due to vitamin B12 (cobalamin) or folate deficiency.^1^ The diagnosis typically relies on clinical presentation, peripheral blood findings (macrocytosis, hypersegmented neutrophils), and confirmatory laboratory testing, including serum B12 and folate levels.^2^ However, a subset of patients presents with discordant laboratory findings, particularly falsely normal or elevated vitamin B12 levels despite clinical and haematological evidence of deficiency.^3,4^

Most clinical laboratories use automated competitive-binding immunoassays to measure vitamin B12. In these assays, patient serum B12 competes with a labelled B12 analogue for binding to intrinsic factor (IF) or IF-like proteins immobilized in the assay system. The measured signal is inversely proportional to the serum B12 concentration. This methodology is vulnerable to analytical interference, particularly in patients with pernicious anaemia.^5^ In pernicious anaemia, autoantibodies against IF (anti-IF antibodies (AIFAs)) not only block the physiological binding of B12 to IF in the gut, leading to malabsorption, but can also bind to IF-based reagents in the assay. This interaction either blocks or mimics B12 binding, resulting in falsely normal or elevated serum B12 values.^5,6^ Consequently, patients may remain undiagnosed or experience delayed treatment, resulting in progressive neurological or haematological complications.

We present a diagnostically challenging case of megaloblastic anaemia with falsely elevated B12 levels due to AIFA interference, emphasizing the importance of clinical correlation, alternative diagnostic strategies, and prompt therapeutic intervention despite seemingly contradictory laboratory results.

## Case presentation

**Figure 1.**
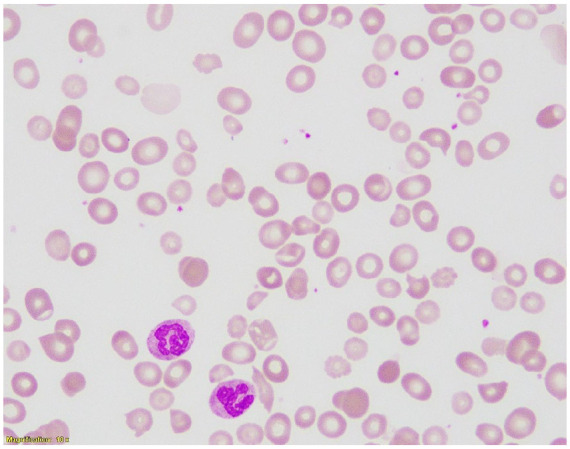
Peripheral blood smear at 100× magnification showing red cell dimorphism and marked anisopoikilocytosis with numerous macro-ovalocytes and hypersegmented neutrophils (two are visible). These findings are characteristic of megaloblastic anaemia and support the diagnosis of vitamin B12 deficiency.

Sixty-four-year-old Arabic woman with no significant past medical history presented with a 3-month history of progressive fatigue, exertional dyspnoea, and palpitations. She denied any neurological symptoms such as paraesthesia, memory impairment, or gait disturbance. There was no history of gastrointestinal surgery, restrictive diet, use of medications known to interfere with B12 absorption (e.g. metformin or proton pump inhibitors), or family history of autoimmune diseases. Physical examination revealed marked pallor, but no evidence of jaundice, glossitis, or neurological deficits. Neurological examination was unremarkable. Initial laboratory investigations showed severe macrocytic anaemia with pancytopaenia and biochemical evidence of ineffective erythropoiesis, including macrocytosis with hypersegmented neutrophils, elevated LDH, indirect hyperbilirubinaemia, and an inappropriately low reticulocyte count (Figure 1 and Table 1). Notably, serum folate was normal, while vitamin B12 levels appeared spuriously elevated (>2000 pmol/L).

**Table 1.** Summary of baseline and 4-week post-treatment laboratory investigations.

Test	Baseline result	Post-treatment result (week 4)	Reference range
Haemoglobin (g/dL)	**8.2**	12.4	12.0–16.0
MCV (fL)	**122**	90	80–100
WBC count (×10^9^/L)	**2.4**	6.1	4.0–11.0
Platelet count (×10^9^/L)	**80**	210	150–400
Reticulocyte count (%)	**0.6**	3.2	0.5–2.5
LDH (U/L)	**680**	180	140–280
Total bilirubin (mg/dL)	**2.1 (indirect 1.0)**	1.1	0.2–1.2
Serum folate (ng/mL)	9.4	–	3.1–17.5
Vitamin B12 (pmol/L)	**>2000**	**>2000**	145–569
Anti-intrinsic factor antibody (U/mL)	**>480.0**		Negative <1.1
Prothrombin time (s)	11		9.4–12.5
Albumin (g/L)	38		35–50
ALT (U/L)	13		0–33
AST (U/L)	11		11–32

ALT: alanine aminotransferase; AST: aspartate aminotransferase; LDH: lactate dehydrogenase; MCV: mean corpuscular volume; WBC: white blood cell.

Bold Signification Values denotes abnormal values.

Given the discrepancy between the clinical picture and the elevated serum B12, differentials such as myelodysplastic syndrome (MDS), liver disease, and assay interference were considered. MDS was unlikely, as the peripheral smear showed classic megaloblastic features rather than dysplastic changes. Liver disease was excluded by the absence of clinical stigmata and normal liver function tests apart from a mild indirect hyperbilirubinaemia (Table 1). The combination of symptomatic anaemia, marked macrocytosis with pancytopenia, smear evidence of megaloblastic haematopoiesis, and biochemical features of ineffective erythropoiesis—along with normal folate—strongly supported vitamin B12 deficiency and justified empirical treatment. AIFA testing returned strongly positive, supporting the diagnosis of pernicious anaemia and explaining the falsely elevated serum B12 due to assay interference. Anti-parietal cell antibodies were not assessed. Methylmalonic acid (MMA) and homocysteine levels were not measured due to unavailability in our laboratory. Gastrointestinal evaluation, including gastroscopy, was not performed during admission but was planned for outpatient follow-up to assess for chronic atrophic gastritis and associated risk of gastric neoplasia.

The patient was commenced on intramuscular vitamin B12 therapy with 1000 µg cyanocobalamin administered daily for 7 days, then weekly for 4 weeks, followed by monthly maintenance doses. She demonstrated rapid clinical and haematological improvement. By week 4 of parenteral vitamin B12 therapy, the patient reported complete resolution of fatigue and exertional symptoms. Laboratory evaluation at the same time demonstrated full haematologic recovery, with normalization of haemoglobin, red cell indices, white blood cell and platelet counts, and an appropriate reticulocyte response (Table 1). Together, these findings confirmed a masked vitamin B12 deficiency diagnosis secondary to pernicious anaemia, initially obscured by AIFA-mediated assay interference.

## Discussion

This case highlights a critical diagnostic pitfall in the evaluation of megaloblastic anaemia—the potential for falsely elevated vitamin B12 levels due to AIFA interference. While the patient presented with classic haematologic features of B12 deficiency, including macrocytic anaemia, hypersegmented neutrophils, and symptomatic pallor, the markedly elevated serum B12 level (>2000 pmol/L) initially caused diagnostic confusion. This paradoxical finding was ultimately explained by the presence of high-titre AIFAs, which interfere with competitive-binding B12 immunoassays and artificially inflate measured values.^5^ Similar cases have been reported in the literature, with studies suggesting that more than 10% of patients with pernicious anaemia may demonstrate this phenomenon of spuriously normal or elevated B12 levels despite true deficiency.^7,8^

The patient’s dramatic haematologic response to parenteral B12 therapy served as both diagnostic confirmation and therapeutic success, underscoring an important clinical principle: when strong clinical evidence suggests B12 deficiency, treatment should not be withheld solely on the basis of seemingly normal or elevated serum levels. This approach is supported by multiple case series demonstrating that AIFA-mediated assay interference can delay diagnosis and treatment if clinicians rely too heavily on serum B12 testing alone.^9–12^ While additional biomarkers such as MMA and homocysteine were unavailable in this case, both are sensitive indicators of functional cobalamin deficiency, and their elevation would have strengthened diagnostic certainty by reflecting intracellular metabolic impairment.^10^ Newer methods, including holotranscobalamin measurement and liquid chromatography–tandem mass spectrometry, provide more reliable assessment as they are less affected by antibody interference, but these remain limited to specialized laboratories and are not yet widely available for routine clinical use.^11–15^

Pernicious anaemia is also associated with chronic atrophic gastritis, which confers an increased risk of gastric adenocarcinoma and type I gastric carcinoids.^16^ In this patient, endoscopic evaluation was deferred during admission, with outpatient upper gastrointestinal endoscopy arranged to confirm the presence of chronic atrophic gastritis and initiate appropriate surveillance. Risk factors for pernicious anaemia include older age, female sex and coexisting autoimmune conditions such as autoimmune thyroid disease, vitiligo and type 1 diabetes mellitus.^17^ Although this patient had no relevant comorbidities or family history, her only risk factors are age and sex.

Finally, this case illustrates the broader implications of laboratory assay limitations. Standard competitive-binding luminescence assays remain vulnerable to interference from endogenous antibodies, particularly in autoimmune conditions such as pernicious anaemia. Until newer assay refinements that neutralize or circumvent antibody interference become routine, clinicians must integrate clinical findings, peripheral smear morphology, and judicious use of available laboratory tests for accurate diagnosis.^5,12–15^ Most importantly, this case reaffirms that laboratory values should always be interpreted in the context of the overall clinical picture—sometimes the numbers do not tell the whole story.

## Conclusion

This case underscores the critical diagnostic pitfall of AIFA-mediated B12 assay interference, which can obscure true deficiency. Clinical judgement and prompt therapeutic intervention remain paramount in managing such patients. Future assays should incorporate methods to neutralize antibody interference, minimizing diagnostic delays.

## Supplemental Material

sj-docx-1-sco-10.1177_2050313X251377211 – Supplemental material for Masked vitamin B12 deficiency in pernicious anaemia: A diagnostic trap due to assay interference—Case report Supplemental material, sj-docx-1-sco-10.1177_2050313X251377211 for Masked vitamin B12 deficiency in pernicious anaemia: A diagnostic trap due to assay interference—Case report by Mohammed Abdulgayoom, Elabbass Abdelmahmuod, Abdulrahman F. Al-Mashdali, Yara R. Abuazab, Aliaa Amer, Ibrahim Ganwo and Shehab Mohamed in SAGE Open Medical Case Reports
