# Supplementary material for: Masked vitamin B12 deficiency in pernicious anaemia: A diagnostic trap due to assay interference—Case report
Source: SAGE Open Med Case Rep. 2025 Sep 15;13:2050313X251377211. doi: 10.1177/2050313X251377211 (PMC12437156; doi:10.1177/2050313X251377211)
Supplement: sj-docx-1-sco-10.1177_2050313X251377211 – Supplemental material for Masked vitamin B12 deficiency in pernicious anaemia: A diagnostic trap due to assay interference—Case report [file sj-docx-1-sco-10.1177_2050313X251377211.docx]

# CARE Checklist (2016) – Author Completed

Manuscript Title: Masked Vitamin B12 Deficiency in Pernicious Anaemia: A Diagnostic Trap due to Assay Interference– Case Report.

Manuscript ID: SOMCR-25-0724

Journal: SAGE Open Medical Case Reports

1. Title: The words 'case report' are included.

2. Key Words: Two to five key words provided (Megaloblastic anaemia, Vitamin B12 deficiency, Pernicious anaemia, Assay interference, Anti-intrinsic factor antibody).

3. Abstract: Unstructured summary provided, covering introduction, case presentation, management, and conclusion.

4. Introduction: Scientific background and rationale explained, including diagnostic pitfall of B12 assays with AIFA interference.

5. Patient Information: De-identified demographic details, clinical presentation, and relevant negatives provided.

6. Clinical Findings: Physical examination findings documented (pallor, no neurological deficits).

7. Timeline: Narrative timeline described in Case Presentation and summarized in text.

8. Diagnostic Assessment: Diagnostic methods, laboratory findings, differential diagnoses, limitations of unavailable MMA/homocysteine tests, and reasoning detailed.

9. Therapeutic Intervention: Intramuscular vitamin B12 regimen described, including dose, frequency, and response.

10. Follow-up and Outcomes: Clinical and hematological improvement at 4 weeks documented, along with planned GI follow-up.

11. Discussion: Strengths, diagnostic pitfalls, literature references, and risk factors addressed. Lessons emphasized.

12. Patient Perspective: Not applicable – patient perspective not obtained.

13. Informed Consent: Written informed consent obtained from patient for publication.

14. References: Appropriate and updated references included.

15. Figures/Tables: Peripheral smear image and consolidated laboratory data table provided with explanatory captions.
